# Mycobacterial receptor, Clec4d (CLECSF8, MCL), is coregulated with Mincle and upregulated on mouse myeloid cells following microbial challenge

**DOI:** 10.1002/eji.201545858

**Published:** 2015-12-08

**Authors:** Bernhard Kerscher, Gillian J. Wilson, Delyth M. Reid, Daiki Mori, Julie A. Taylor, Gurdyal S. Besra, Sho Yamasaki, Janet A. Willment, Gordon D. Brown

**Affiliations:** ^1^ Institute of Medical SciencesUniversity of AberdeenForesterhillAberdeenUK; ^2^ Division of Molecular Immunology, Medical Institute of BioregulationKyushu UniversityKyushuJapan; ^3^ School of BiosciencesUniversity of BirminghamBirminghamUK

**Keywords:** C‐type lectin receptor, CLECSF8, CLEC4E, CLEC4N, Innate immunity, MCL, *Mycobacterium bovis* BCG

## Abstract

The C‐type lectin receptor (CTLR), Clec4d (MCL, CLECSF8), is a member of the Dectin‐2 cluster of CTLRs, which also includes the related receptors Mincle and Dectin‐2. Like Mincle, Clec4d recognizes mycobacterial cord factor, trehalose dimycolate, and we recently demonstrated its key role in anti‐mycobacterial immunity in mouse and man. Here, we characterized receptor expression in naïve mice, under inflammatory conditions, and during *Mycobacterium bovis* BCG infection using newly generated monoclonal antibodies. In naïve mice, Clec4d was predominantly expressed on myeloid cells within the peritoneal cavity, blood, and bone marrow. Unexpectedly, basal expression of Clec4d was very low on leukocytes in the lung. However, receptor expression was significantly upregulated on pulmonary myeloid cells during *M. bovis* BCG infection. Moreover, Clec4d expression could be strongly induced in vitro and in vivo by various microbial stimuli, including TLR agonists, but not exogenous cytokines. Notably, we show that Clec4d requires association with the signaling adaptor FcRγ and Mincle, but not Dectin‐2, for surface expression. In addition, we provide evidence that Clec4d and Mincle, but not Dectin‐2, are interdependently coregulated during inflammation and infection. These data show that Clec4d is an inducible myeloid‐expressed CTLR in mice, whose expression is tightly linked to that of Mincle.

## Introduction

The Dectin‐2 family of C‐type lectin receptors (CTLRs) encoded upstream of the murine and human NK gene cluster consists of CLECSF8 (Clec4d), Mincle (Clec4e), Dectin‐2 (Clec4n), DCIR (Clec4a), DCAR (Clec4b), and BDCA‐2 (Clec4c). All these receptors are type II transmembrane receptors possessing a single C‐type lectin‐like domain (CTLD), a stalk and transmembrane region, and, with the exception of DCIR, a short cytoplasmic tail 1. These receptors recognize a diverse range of endogenous and exogenous ligands, and can function as pattern recognition receptors for several classes of pathogens including fungi, bacteria, and parasites, driving both innate and adaptive immune responses 1.

Of interest here is Clec4d (also called macrophage C‐type lectin (MCL), Dectin‐3, CLEC6, CLECSF8), a CTLR identified over 15 years ago in the Gordon laboratory following a screen of a murine differential display library for macrophage‐specific genes 2. Subsequent analysis of protein expression in human and rat, however, has shown that Clec4d can also be expressed by other myeloid cell types, including neutrophils and monocytes 3, 4. Like Mincle and Dectin‐2, surface expression of Clec4d requires association with the signaling adaptor FcRγ, which also mediates downstream signaling through the Syk/CARD9 pathway 1, 5. Unlike the other Dectin‐2 family members, however, intracellular retention of Clec4d, in the absence of adaptors, is mediated by the C‐type lectin domain and not the transmembrane region 3. This is likely to be due to the ability of Clec4d to form functional receptor complexes with Dectin‐2 and Mincle 6, 7. In fact, Clec4d was found to be required for the induction and surface expression of Mincle 5, 7.

The CTLD of Clec4d lacks the conserved amino acids normally associated with carbohydrate recognition and, unlike the other Dectin‐2 family receptors, is unable to recognize unconjugated sugars 3. However, Clec4d possesses a shallow hydrophobic region on its surface, enabling it to bind glycolipids, including mycobacterial cord factor (trehalose dimycolate, TDM) 5, 8. We recently demonstrated that Clec4d plays a key role in anti‐mycobacterial host defense, acting as a nonopsonic receptor for mycobacteria on pulmonary leukocytes 9. Loss of this C‐type lectin receptor in mice resulted in exacerbated inflammation, higher mycobacterial burdens, and increased mortality 9. Furthermore, a polymorphism of Clec4d in humans, which caused reduced surface expression of the receptor, is associated with increased susceptibility to pulmonary tuberculosis 9. Given the importance of the murine model for further study of this receptor in anti‐mycobacterial immunity, we describe here the characterization of Clec4d expression during resting and inflammatory conditions, and during infection with *Mycobacterium bovis* Bacillus Calmette–Guérin (BCG) in the mouse. We have also further investigated the codependent relationship of Clec4d expression with Mincle and Dectin‐2.

## Results

### Generation of anti‐mClec4d monoclonal antibodies

**Figure 1:**
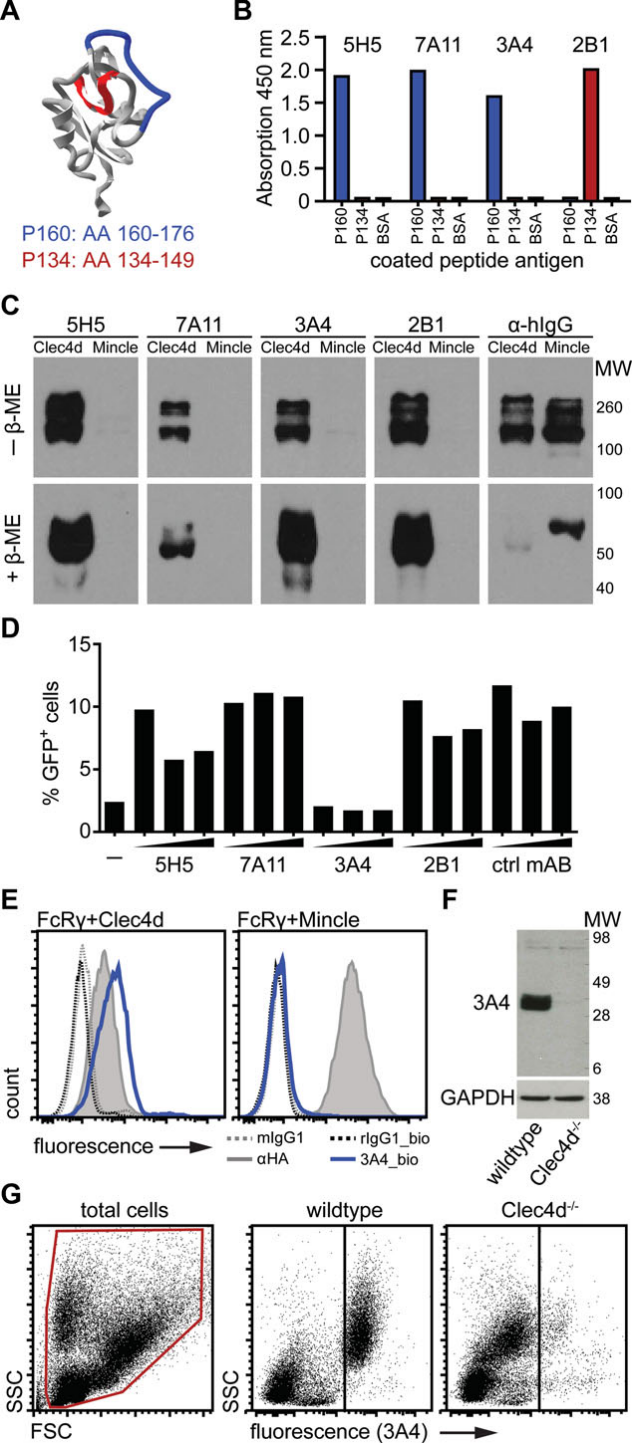
Generation and characterization of novel mClec4d monoclonal antibodies. (A) Protein model (visualized using spdbv v4) of Clec4d indicating the location of the KLH‐conjugated peptide immunogens (P160, blue, and P134, red, corresponding to amino acid (AA) 160–176 and AA134‐149, respectively). (B) BSA‐conjugated peptide or BSA alone were coated on plates and detected with selected antibody clones by ELISA to type their peptide specificity, as indicated. Data shown are from one experiment with one sample per condition. (C) Western blot of mClec4d‐Fc (Clec4d) and mMincle‐Fc (Mincle), separated by SDS‐PAGE under nonreducing (–β‐ME) or reducing (+β‐ME) conditions, probed with the various antibody clones or anti‐human IgG (α‐hIgG), as indicated. Blot from a single experiment. (D) Effect of various mAB clones, as indicated, on GFP expression following BCG stimulation of Clec4d‐expressing reporter cells, unstimulated cells; ctrl mAB, unrelated control rat‐anti mouse mAB. Data from one representative experiment with one sample per condition. (E) Flow cytometry analysis of NIH‐3T3 cells expressing FcRγ and HA‐tagged Clec4d or HA‐tagged Mincle, stained with anti‐HA, biotinylated‐3A4 (3A4_bio), or isotype controls, as indicated. Data are from a single experiment. (F) Western blot of 50 μg RIPA‐lysates from wild type and Clec4d^−/−^ BMM stimulated with LPS for 16 h, probed with 3A4 (top) and following stripping with anti‐GAPDH (bottom). Data from a single experiment. (G) Flow cytometric analysis of PBL from wild type and Clec4d^−/−^ mice, stained with 3A4. Plots are representative of three experiments.

To investigate expression of mClec4d, we generated and characterized novel mABs specific for this receptor using two keyhole limpet hemocyanin (KLH)‐linked Clec4d peptides (P160 and P134) located within unique regions of the extracellular domain (Fig. 1A). Attempts using the intact CTLD of Clec4d did not generate specific mABs (data not shown). Rats were immunized with the peptides and hybridomas generated and screened as described in materials and methods. We selected four clones that bound either BSA‐conjugated Clec4d peptide by ELISA (Fig. 1B). All clones detected a soluble chimeric protein containing the CTLD of Clec4d fused to the Fc‐portion of human IgG1, Clec4d‐Fc 3, but not the closely related Mincle‐Fc 5, by Western blot under reducing and nonreducing conditions (Fig. 1C). To assess the potential of these clones to be used as blocking antibodies, we made use of the Clec4d reporter cell system that induces GFP expression upon stimulation with mycobacteria 5, 9. We found that pretreatment of the cells with the mAB clone 3A4 abolished reporter cell activation induced by *M. bovis* BCG making it a potentially valuable tool for in vivo receptor blocking experiments in the future (Fig. 1D). The 3A4 antibody further stained Clec4d but not Mincle transduced NIH‐3T3 fibroblast cells (Fig. 1E). To further confirm specificity, we compared 3A4 staining of primary wild type and Clec4d‐deficient cells. Bone marrow‐derived macrophage (BMM)‐lysates probed by Western blot revealed a band of expected size (Fig. 1F) and analysis of murine PBL by flow cytometry using 3A4 (Fig. 1G) revealed staining of wild type but not Clec4d‐deficient cells. Thus 3A4 was used in all further experiments.

### Characterization of mClec4d expression in the naïve mouse

**Figure 2:**
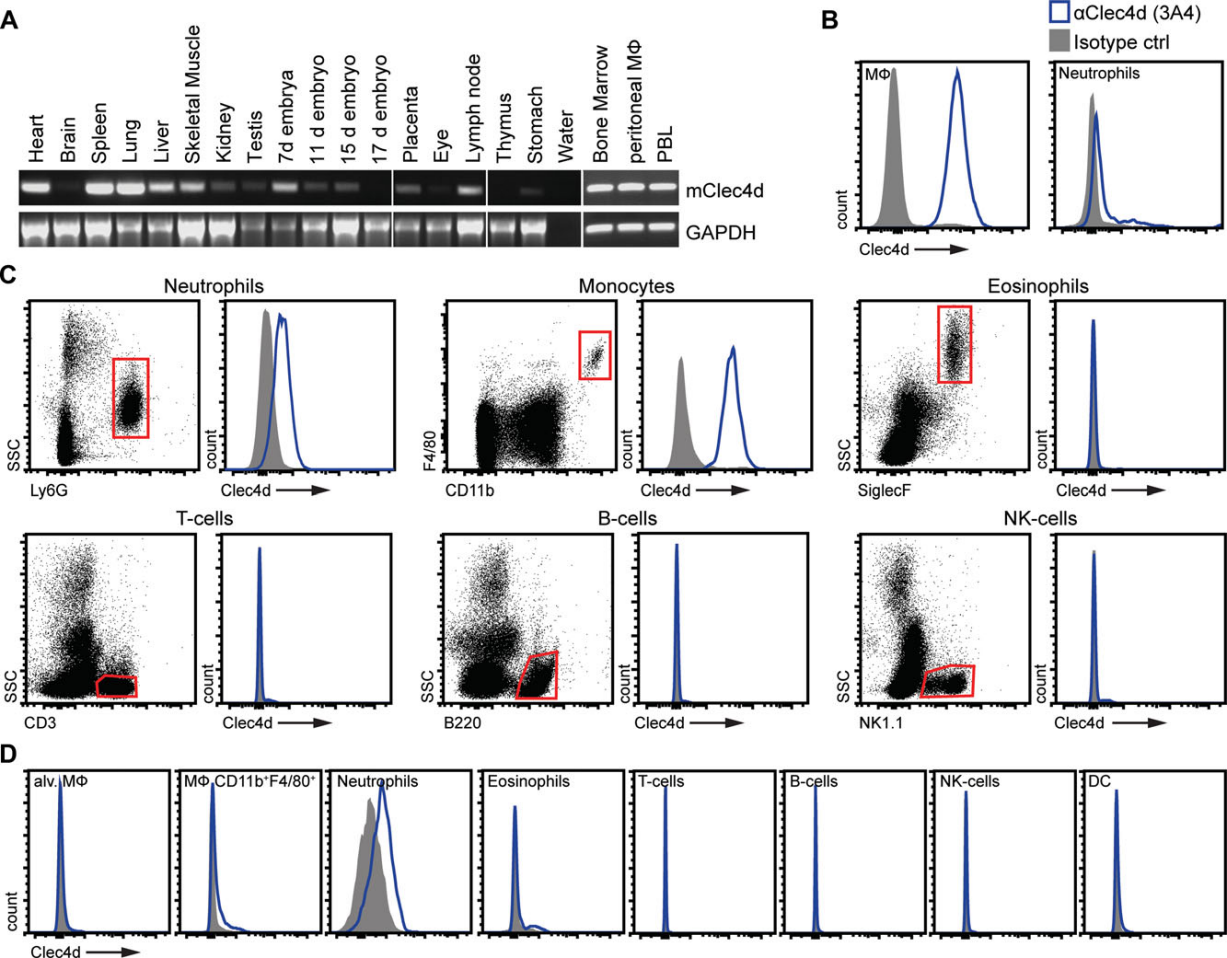
Clec4d is expressed on myeloid cells. (A) RT‐PCR analysis of Clec4d expression in cells and tissues, as indicated. GAPDH served as a housekeeping control. Data from a single experiment. (B–D) Representative flow cytometry plots of Clec4d protein expression on (B) resident peritoneal cells, (C) PBL, and (D) lung. (C) An example of the gating strategies used is shown for PBL. Gray filled histograms show IgG1‐stained cells while open blue histograms show Clec4d (3A4) stained cells. (B–D) Data shown are representative of at least three animals representative of at least two experiments performed.

To characterize the cellular expression of Clec4d in mice, we firstly determined its mRNA expression profile in various tissues by reverse transcription (RT) PCR (Fig. 2A). Similar to human Clec4d 3, we found mClec4d transcript to be widely expressed in tissues including liver, muscle, embryo, placenta, and lymph node with strong expression in heart, spleen, lung, bone marrow, PBL, and resident peritoneal macrophages. Based on these expression patterns, we then characterized Clec4d protein expression on cells within various transcript‐positive and ‐negative tissues using our novel mAB, 3A4.

We first analyzed Clec4d expression on cells harvested by peritoneal lavage from naïve C57BL/6 mice, as the receptor was originally cloned from these cells by differential display PCR 2. Consistent with the mRNA expression profile (Fig. 2A and 2) we found Clec4d to be highly expressed on resident F4/80^+^CD11b^+^ peritoneal macrophages (Fig. 2B). In fact, expression of Clec4d on these cells was the highest compared to all other cells/tissues analyzed in naïve animals. Low levels of Clec4d expression were also detected on the surface of Ly6G^+^ resident peritoneal neutrophils. Within PBL, Clec4d was detected on Ly6G^+^ neutrophils and F4/80^+^CD11b^+^ monocytes, while this receptor was not detectable on the surface of eosinophils, T cells, B cells, and NK cells (Fig. 2C). Similarly, we found expression on splenic cells and in the bone marrow, but no expression was detected on the surface of cells in the lymph nodes despite observing Clec4d transcripts in this tissue (Fig. 2A and Supporting Information Fig. 1A–C). Consistent with our RT‐PCR analysis, no significant expression of Clec4d was observed in eye tissue (Fig. 2A and Supporting Information Fig. 1D).

Since Clec4d plays a crucial role in the innate immune response to pulmonary *Mycobacterial* infections 9, we next characterized Clec4d expression in the lung. Unexpectedly, Clec4d was not detected on the surface of SiglecF^+^ alveolar macrophages and was only expressed at low levels on Ly6G^+^ neutrophils and marginal levels on CD11b^+^F4/80^+^ macrophages (Fig. 2D). No Clec4d expression was detected on the other pulmonary leukocytes examined (Fig. 2D).

### Modulation of Clec4d and Mincle expression on macrophages

**Figure 3:**
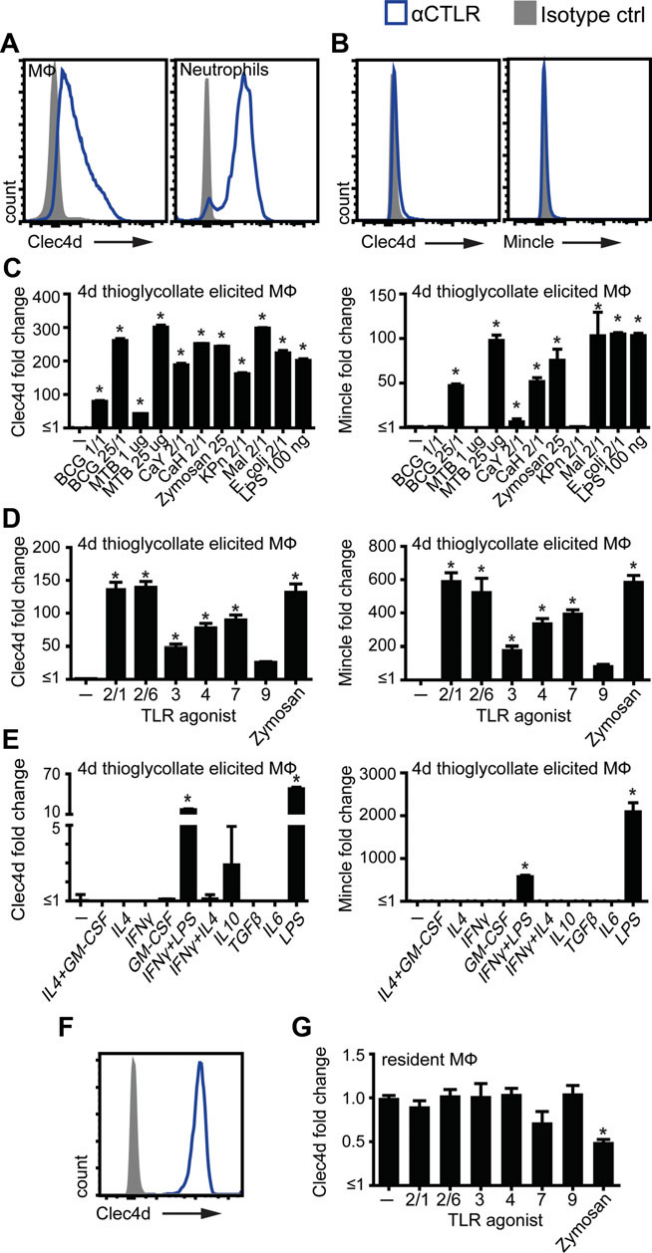
Modulation of Clec4d and Mincle expression by microbial stimuli. (A–F) Flow cytometric analysis of receptor expression on mouse peritoneal cells. (A) Peritoneal recruited cells 4 days after i.p. thioglycollate injection were analyzed for Clec4d expression (representative of >3 mice from two experiments). Plated thioglycollate‐elicited peritoneal macrophages were analyzed for Clec4d and Mincle expression under (B) resting conditions, which indicates the baseline for further stimulations (representative of >3 experiments) or after stimulation for 16 h with (C) microbial agents: BCG, *M. bovis* BCG; MTB, *M. tuberculosis*; CaY, *C. albicans* yeast; CaH, *C. albicans* hyphae; KPn, *K. pneumoniae*; Mal, *Malassezia* (representative of a single experiment), (D) TLR agonists (pooled data from two experiments performed in duplicates) or (E) cytokines (representative of a single experiment performed in duplicates). (F) Resident peritoneal cells were plated and (G) stimulated with TLR agonists for 16 h followed by Clec4d‐expression analysis by flow cytometry (pooled data from two experiments performed in duplicates). All data shown as mean + SD for single experiments and mean + SEM for pooled experiments of the indicate number of samples. Statistical significance was determined by ANOVA and Dunnett's multiple‐comparison posttests; *p < 0.05.

Expression of CTLRs can be modulated by inflammatory conditions 1, so we next assessed expression of Clec4d on 4‐day thioglycollate‐elicited peritoneal inflammatory cells. The highest levels of Clec4d expression were detected on Ly6G^+^ neutrophils, which represented less than <5% of the population at this time point (Fig. 3A). We also detected Clec4d expression on elicited CD11b^+^F4/80^+^ macrophages, but not at the levels seen on resident macrophages (compare to Fig. 2B). Moreover, isolation and culturing these cells in vitro led to substantially reduced expression of this receptor (Fig. 3B), similar to what we had observed with human cells 3. Since Clec4d and Mincle are closely related receptors, sharing the same ligand, we decided to include this receptor in our investigation. Similarly to Clec4d, Mincle expression was absent on cultured thioglycollate‐induced macrophages (Fig. 3B). These expression levels formed the baseline for further investigation of the effect of microbial stimuli on these plated macrophages. Notably, there was a robust and dose‐dependent upregulation of Clec4d and Mincle on these cells in response to BCG and heat‐killed *Mycobacterium tuberculosis*, as well as to fungi and Gram^−^ bacteria (Fig. 3C). Similar results were obtained for BMM (Supporting Information Fig. 2).

These observations led us to investigate if expression of these receptors on macrophages is modulated by defined biological agents. We first tested a variety of TLR agonists, which lead to substantial upregulation of Clec4d and Mincle expression on cultured thioglycollate‐elicited cells (Fig. 3D). In contrast, there was no significant effect on Clec4d and Mincle expression following stimulation with a variety of cytokines, although costimulation with IFNγ significantly reduced LPS‐induced receptor expression (Fig. 3E). The very high Clec4d expression on resident macrophages was retained after plating (Fig. 3F) and treatment of these resident macrophages in vitro with TLR‐agonists did not significantly change Clec4d expression, although there was a slight but significant reduction upon treatment with zymosan (Fig. 3G).

### Clec4d expression is coregulated with Mincle, but not Dectin‐2

**Figure 4:**
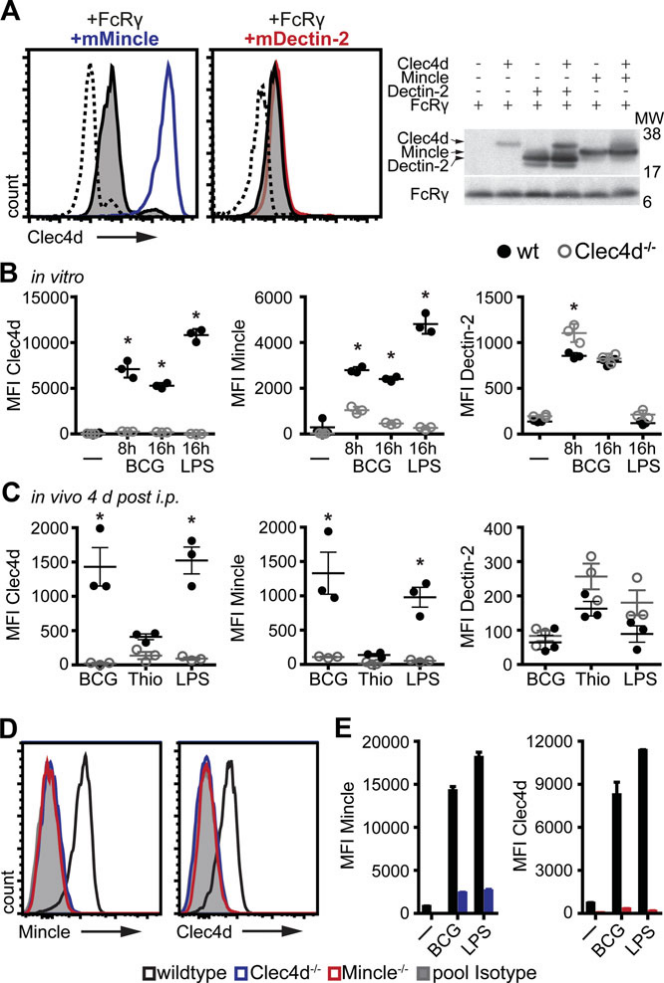
Clec4d forms a complex with Mincle, but not Dectin‐2. (A) NIH‐3T3 fibroblast cells were transduced with FcRγ, Clec4d, and Mincle or Dectin2, as indicated, followed by flow cytometric analysis of Clec4d surface expression (left). Representative histograms of four independent experiments performed on individual samples are shown: dotted histograms, FcRγ alone; gray filled histograms, FcRγ with Clec4d; blue and red histograms, Clec4d expression in the presence of Mincle and Dectin‐2, respectively. Receptor protein expression was confirmed by Western blot using 25 μg of RIPA cell lysates probed with anti‐HA or anti‐FcRγ followed by peroxidase conjugated anti‐mouse or anti‐rabbit, respectively (right). (B) Thioglycollate elicited macrophages from wild type or Clec4d^−/−^ mice (*n* = 3) were stimulated as indicated and Clec4d (3A4), Mincle (4A9), and Dectin‐2 (11E4) expression assessed by flow cytometry and represented as MFI. (C) Wild type or Clec4d^−/−^ mice (*n* = 3) were injected with immunomodulators intraperitoneally and cells harvested 4 days later by peritoneal lavage followed by flow cytometric analysis of CTLR expression. (B, C) Each symbol represents an individual mouse and bars represent mean + SEM of single experiments representative of one (B) or two (C) experiments performed. Statistical significance was determined by ANOVA and Dunnett's multiple‐comparison posttests, *p < 0.05. (D) PBL from naïve wild‐type (black histograms), Clec4d^−/−^ (blue histograms), and Mincle^−/−^ (red histograms) mice were analyzed for receptor expression by flow cytometry. Histograms represent Clec4d (3A4) and Mincle (4A9) expression on Ly6G+ PMNs compared to IgG1 isotype staining (gray filled histograms). Data shown are from a single experiment. (E) BMM from wild type (black bars), Clec4d^−/−^ (blue bars) or Mincle^−/−^ mice (red bars) were left untreated or treated with *M. bovis* BCG or LPS for 16 h and receptor expression assessed by flow cytometry. Data are shown as mean + SD from a single experiment performed in duplicates.

Recent papers have suggested that Clec4d forms functional heterodimers with other CTLRs, particularly Mincle 7 and Dectin‐2 6. To explore this in more detail, we made use of the mouse fibroblast cell line NIH‐3T3 in which Clec4d is retained intracellularly 3 and retrovirally cotransduced these cells with FcRγ along with Mincle or Dectin‐2. In agreement with previous reports, we found that cotransduction with FcRγ alone was able to promote moderate levels of Clec4d surface expression (Fig. 4A) 5. Strikingly, however, additional cotransduction of Mincle, but not Dectin‐2, led to a substantial increase of Clec4d surface expression on these cells (Fig. 4A) while total Mincle and Dectin‐2 protein levels were comparable (Fig. 4A and Supporting Information Fig. 3A). This suggests that regulation of surface expression occurs at a posttranslational level and shows that Clec4d requires Mincle, but not Dectin‐2, for optimal expression at the cell surface. This is consistent with our observation that expression of Mincle mirrored that of Clec4d in isolated thioglycollate–elicited macrophages and in BMM following stimulation with microbial and TLR agonists and cytokines (Fig. 3B–E).

### Clec4d induces Mincle‐expression in vivo

Our data suggest that Clec4d expression depends on Mincle, but we have also previously shown that the induction of Mincle requires Clec4d 5. To investigate the effects of Clec4d‐deficiency, we examined Mincle expression on a variety of cells types in naïve

wild type and in Clec4d‐deficient mice. Notably, Mincle expression closely mirrored Clec4d‐expression in resident peritoneal macrophages, and was lost in the absence of Clec4d (Supporting Information Fig. 3B). As these data suggest an interdependent coregulation of both receptors, but not Dectin‐2, we further examined this phenomenon in isolated primary macrophages in vitro and on inflammatory cells in vivo. Thioglycollate‐elicited macrophages from C57BL/6 wild‐type and Clec4d^−/−^ mice were stimulated with *M. bovis* BCG and LPS in vitro and receptor expression of Clec4d, Mincle, and Dectin‐2 analyzed by flow cytometry. All treatments led to a robust upregulation of expression of these three receptors on wild‐type cells (Fig. 4B). However, stimulated Clec4d^−/−^ macrophages failed to robustly induce Mincle, while expression of Dectin‐2 was largely unaffected (Fig. 4B). Similarly, in Clec4d^−/−^ mice in vivo, Mincle expression was impaired on thioglycollate‐, LPS‐, and BCG‐induced inflammatory peritoneal cells at 1 and 4 days after i.p. injection (Fig. 4C and Supporting Information Fig. 3C). In contrast, Clec4d‐deficiency had no effect on Dectin‐2 expression (Fig. 4C and Supporting Information Fig. 3C).

### Mincle is required for Clec4d surface expression

We have shown that Clec4d is required to induce Mincle expression in vitro and in vivo. Yet, our initial experiments coexpressing Mincle and Clec4d in NIH‐3T3 cells (Fig. 4A) suggested that Mincle was reciprocally required for the surface expression of Clec4d. To investigate this interdependence further, we made use of Mincle‐deficient mice. As we had observed on resident peritoneal macrophages (Supporting Information Fig. 3B), the expression of Mincle and Clec4d on peripheral blood neutrophils or CD11b^+^ bone marrow cells from naïve mice closely mirrored each other. As before, Mincle expression was lost in the absence of Clec4d (Fig. 4D and Supporting Information Fig. 4) and conversely, Clec4d expression was absent on Mincle‐deficient cells (Fig. 4D and Supporting Information Fig. 4). Since Mincle was essential for Clec4d expression under naïve conditions, we further investigated whether Mincle was required for Clec4d upregulation following microbial challenge. Wild‐type BMM robustly upregulated Clec4d and Mincle expression in response to BCG and LPS (Fig. 4E). However, the lack of Mincle resulted in a failure to significantly upregulate Clec4d and vice versa (Fig. 4E). Taken together, these data demonstrate that Mincle and Clec4d are coregulated and dependent on each other, forming a functional receptor complex required for surface expression.

### Clec4d expression during pulmonary mycobacterial infection

**Figure 5:**
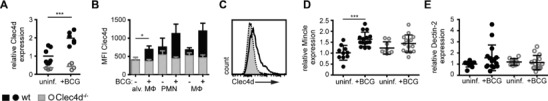
Coregulation of Clec4d and Mincle during pulmonary *M. bovis* BCG infection. CTLR expression on CD45^+^ pulmonary leukocytes in naïve wild type and Clec4d^−/−^ mice and 48 h following infection with *M. bovis* BCG were analyzed by flow cytometry. (A) CD45^+^ and (B) population‐specific cellular expression levels of Clec4d were determined by flow cytometry. Shown are pooled data from two independent experiments, *n* = 6–9 mice per group from both experiments. (C) Representative histogram of Clec4d expression on alveolar macrophages following infection as above. Black dotted histogram, wild‐type naïve; gray filled histogram, Clec4d^−/−^ +BCG; solid black histogram, wild‐type +BCG. (D) Expression levels of Mincle on CD45^+^ cells. Shown are pooled data from three experiments, *n* = 9–15 mice per group. (E) Expression levels of Dectin‐2 on CD45^+^ cells. Shown are pooled data from three experiments, *n* = 9–15 mice per group in total. (A, D, E) Each symbol represents an individual mouse and bars represent mean + SEM. Statistical significance was determined by Student's *t*‐test, *p < 0.05, ***p < 0.001.

We recently discovered that Clec4d plays a key role in anti‐mycobacterial immunity by facilitating bacterial clearance by pulmonary leukocytes 9. As the levels of Clec4d expression on pulmonary leukocytes were relatively low in naïve mice (Fig. 2D), expression of this receptor in the lung was examined following infection with *M. bovis* BCG. Two days post intratracheal infection with BCG, there was a significant increase in total Clec4d expression on CD45^+^ pulmonary leukocytes compared to uninfected control mice (Fig. 5A). In fact, infection with BCG led to increases in Clec4d expression on all myeloid cell populations examined, including a significant increase on alveolar macrophages (Fig. 5B, C and Supporting Information Fig. 5). Similarly, expression of Mincle was robustly induced in wild‐type mice following infection with BCG (Fig. 5D). In contrast to our results with peritoneal cells (Figs. 3 and 4), expression of Mincle was detectable on pulmonary cells isolated from naïve Clec4d^−/−^ mice (Fig. 5D). However, in contrast to wild‐type mice, infection of the knockout animals with BCG did not lead to an increased expression of Mincle (Fig. 5D). Correspondingly, plated alveolar macrophages from naïve mice required coexpression of Clec4d to robustly upregulate Mincle in response to BCG and LPS in vitro (Supporting Information Fig. 5). Expression of Dectin‐2 was unaltered following BCG infection in wild‐type and Clec4d‐deficient mice (Fig. 5E).

## Discussion

There is increasing appreciation that CTLR play key roles in protective immunity to a variety of pathogens 10. Notably, mice lacking the downstream signaling adaptor, CARD9, are extremely susceptible to infections with fungi and *Mycobacteria* 11. Several CTLRs have subsequently been implicated in mycobacterial recognition including Mincle, Dectin‐2, and Clec4d 9, 10, 12. Although Mincle‐deficiency led to increased *M. bovis* BCG lung burdens, of these receptors, only Clec4d has so far been found to play a nonredundant role during infection in vivo resulting in significant mortality 9, 13. Thus given the importance of this receptor, and the mouse as a model of mycobacterial infection 14, we characterized the expression of Clec4d in naïve mice, under inflammatory conditions and during mycobacterial infection.

In naïve animals, we detected Clec4d expression on myeloid cells in a variety of tissues, particularly on cells of the monocyte/macrophage and neutrophil lineages. Similar expression profiles have been described in human and rat 3, 4. The highest levels of expression were detected on the surface of resident peritoneal macrophages, consistent with transcriptional analysis performed during the initial characterization of the receptor 2. Surprisingly given its role in anti‐mycobacterial immunity, and in contrast to rat 4, Clec4d was poorly expressed by pulmonary myeloid cells and not detectable on alveolar macrophages. However, pulmonary infection with *M. bovis* BCG led to upregulation of this receptor on the surface of pulmonary leukocytes including alveolar macrophages. These increases in Clec4d expression levels within the lung were lower than expected, albeit significant for alveolar macrophages. However, we showed previously that a complete loss of Clec4d (and therefore potentially Mincle) resulted in a profound defect in the innate response toward mycobacterial infection in a mouse model, underlining the importance and relevance of Clec4d expression in this tissue 9. Importantly, this increased Clec4d expression during infection is reflective of the transcriptional upregulation of this receptor that we had observed in patients suffering from pulmonary tuberculosis 9.

The induction of peritoneal inflammation using thioglycollate, LPS or *M. bovis BCG* in our mouse models led to the recruitment of Clec4d‐expressing inflammatory macrophages and neutrophils. Culture of inflammatory macrophages in vitro, however, led to a complete loss of Clec4d expression, similar to effect observed following culture of human monocytes 3. Stimulation of the cultured murine cells with a variety of intact and purified microbial agonists, but not with cytokines, was able to increase surface expression of Clec4d. In contrast, expression of the receptor on resident macrophages was largely unaffected following culture in vitro or following stimulation with microbial agonists. These observations suggest that the mechanisms involved in the regulation of Clec4d‐expression differ between resident and inflammatory macrophages. While the induction of this receptor on inflammatory cells makes physiological sense, in terms of its role in antimicrobial immunity, why resident macrophages have such a high level of expression is currently unclear. It is possible that like many other C‐type lectins, Clec4d recognizes endogenous ligand(s) and plays a homeostatic role that has yet to be determined 1.

Clec4d has been shown to form functional heteromeric complexes with Dectin‐2 and Mincle 6, 7. Clec4d has also previously been shown to be required for the induction of Mincle following stimulation with TDM 5, through CARD9‐dependent NFκB activation 15. While our data clearly support an interaction between Clec4d and Mincle, we found no evidence that Dectin‐2 was necessary for surface expression of Clec4d, nor was expression of Dectin‐2 changed in the absence of Clec4d. Mincle, but not Dectin‐2, was able to transport Clec4d to the surface of transfected cells. Furthermore, Mincle was essential for steady‐state Clec4d expression on primary cells and its upregulation in response to microbial stimuli in vitro. This is corroborated by a recent study showing that Mincle‐deficient bone marrow‐derived DCs fail to upregulate Clec4d‐expression in response to LPS 16. Moreover, we found that the expression of Mincle, but not Dectin‐2, was regulated in a manner similar to that of Clec4d, following microbial stimulation in vitro and in vivo. In addition, deficiency of Clec4d correlated with a loss of Mincle expression on peritoneal inflammatory cells, while there was no effect on expression of Dectin‐2. Intriguingly, Mincle was expressed in the naïve lung in Clec4d‐deficient animals, consistent with our observation that surface expression of this receptor only requires the FcRγ adaptor. However, in the absence of Clec4d, expression of Mincle is not upregulated following mycobacterial infection. Thus our data suggest that there is an interdependent coregulation of both receptors for expression at the cell surface. As this interdependence is likely to play an important role in anti‐mycobacterial immunity, future work should be aimed at determining the underlying mechanisms.

## Materials and methods

### Animals

C57BL/6 and Clec4d^−/−^ 3 mice, sex, and age matched as required, were obtained from specific pathogen‐free facilities at the University of Aberdeen. All animal experiments were in agreement with the animal care and welfare protocols approved by the University of Aberdeen (project licences 60/4007 and 70/8073).

### Strains, growth conditions, and infections

*M. bovis* BCG strain Pasteur and a GFP‐expressing derivative 17 were grown on Middlebrook 7H10 agar plates containing 10% ADC (BD), or Middlebrook 7H9 broth containing 10% ADC and 0.05% Tween 80 (Sigma). For in vitro stimulation experiments, *E. coli* and *K. pneumoniae* were grown in nutrient broth, *C. albicans* in yeast‐peptone‐dextrose broth overnight at 37ºC. Fungal hyphae were induced in PBS with 10% foetal bovine serum (Gibco) at 37ºC until hyphal morphology was observed microscopically, usually within 4 h.

To assess cell recruitment and receptor expression during pulmonary infection, 5 × 10^5^ colony forming units BCG were administered intratracheally (i.t.) to anesthetized mice. Bronchoalveolar lavages were performed 2 days after infection with PBS containing 5 mM EDTA (Gibco). Sterile peritonitis was induced by injecting 1 mL thioglycollate (BD), 1 × 10^7^ heat‐killed BCG or 1 μg LPS (Sigma) i.p. and cells were harvested by peritoneal lavage with PBS 5 mM EDTA after 1‐4 days, as stated in the text.

### Cells, cell lines, and stimulation

NFAT‐GFP reporter cells, cotransfected with mClec4d and FcRγ, were described previously 5. NIH‐3T3 mouse fibroblast cell lines coexpressing FcRγ, mClec4d_HA, and mMincle_HA or mDectin‐2_his_6_ or mDectin‐2_HA were generated by retroviral transduction as described previously 3, 18. Receptor expression was confirmed by Western blot following conventional protocols using antibodies against the HA‐tag (HA.11 clone 16B12, Covance) and FcRγ (06‐727, Millipore) followed by appropriate peroxidase‐conjugated secondary antibodies (Jackson). Cell lines were generated at least twice and used as nonclonal populations to reduce founder effects. BMMs were generated from murine bone marrow cells by addition of L929 supernatant (M‐CSF), as described previously 19. The effect of TLR agonists (Invivogen), cytokine treatment (R&D, Miltenyi), and zymosan (Sigma) on receptor expression was performed as described previously 20, 21. Generally, cells were seeded at 4 × 10^5^ per well in 24‐well plates and cultured overnight, followed by three washes before the addition of stimuli for 16 h. Then, cells were analyzed by flow‐cytometry, as described below. For each treatment cells were stained with anti‐receptor mABs and the respective isotype controls and fold change of expression calculated as described before 21: the mean fluorescence of isotype controls were subtracted from the mean fluorescence of anti‐receptor stained cells and the resulting mean fluorescence of treated samples divided by the mean fluorescence of the untreated controls.

### RT‐PCR analysis

Template for RT PCR was a commercial tissue cDNA panel (Clontech). For bone marrow, blood, and peritoneal macrophages, mRNA was isolated using TRI reagent (Applied Biosystems) and RNeasy kit (Qiagen), followed by cDNA synthesis with SuperScript III First‐Strand SuperMix (Invitrogen) according to manufacturers’ instructions. Clec4d mRNA expression was analyzed using standard PCR protocols with primers mClec4d_F (5’AGGTACTTGGACCTGCTGTCCTGTAGC), mClec4d_R (5’TCCCCCTTTTCCCAGAATACCGTGTG), and Clontech GAPDH or mGAPDH_F (5’CCAGGTTGTCTCCTGCGACTT) and mGAPDH_R (5’CCTGTTGCTGTAGCCGTATTCA) primers.

### Generation of monoclonal antibodies

To generate Clec4d mABs, Sprague Dawley rats were immunized with KLH‐conjugated Clec4d peptides (GenScript), as described in the text, splenocytes harvested, and fused to Y3 rat myeloma cells as described before 22. Hybridoma supernatants were screened by ELISA using BSA‐conjugated Clec4d peptides and by flow cytometry using Clec4d transduced cell lines. Monoclonality was attained by limiting dilution. Detection of purified Clec4d‐Fc and Mincle‐Fc or endogenous Clec4d and GAPDH (Clone mAbcam 9484) in BMM by Western blot followed conventional protocols using appropriate HRP‐linked secondary antibodies (Jackson) 3, 18.

### Flow cytometry analysis

Receptor expression was analyzed by flow cytometry using BD LSRII, Fortessa, and Array flow cytometers as described previously 3 and analyzed using FlowJo vX.0.6. Cell populations were identified using the following antibodies purchased from BD or Abd Serotec: CD45 (104), CD11b (M1/70), CD11c (HL3), Ly‐6G (1A8), SiglecF (E50‐2440), F4/80 (Cl:A3‐1), CD3 (145‐2C11), CD4 (RM4‐5), CD8 (53‐6.7), NK1.1 (PK163), CD49b (DX5), B220 (RA3‐6B2), 7/4 (MCA771). HA‐tagged receptors were detected using Mouse anti‐HA.11 (Covance) and anti‐mouse PE (Jackson). We used in‐house developed antibodies against Clec4d (3A4, described in the text), Mincle (Clone 4A9) 5, and Dectin‐2 (Clone 11E4) 23 in conjunction with anti‐rat PE/Cy5 secondary antibodies (Jackson) or streptavidin APC/PE‐CF594 (Invitrogen/BD). Monocytes/macrophages were defined as CD11b^+^/F4/80^+^, alveolar macrophages as CD11c^+^/SiglecF^+^, PMN as CD11b^+^/Ly6G^+^, eosinophils as SSC^hi^/SiglecF^+^, dendritic cells as CD11c^+^, lymphocytes were defined by SSC and expression of CD3 (T cells), B220 (B cells), and NK1.1 or CD49b (NK cells).

Tissues were harvested and dissociated in the presence of liberase and DNase (Roche) (lung, eye), or directly passed through cell strainers (spleen, bone marrow, lymph nodes), red blood cells lysed using FACS Lyse (BD), followed by FACS analysis performed as described previously 3. Briefly, cells were stained in FACS wash (PBS with 0.5% BSA, 10 mM EDTA, 2 mM sodium azide) with 5% rabbit serum (FACS block) and where appropriate 4 μg/mL 2.4G2 at 4ºC. Following washing, cells were incubated with the respective secondary antibody in FACS block at 4ºC. After further washing, cells were fixed with 1% paraformaldehyde in FACS wash and analyzed by flow cytometry.

### Statistics

Statistical analyses were performed using ANOVA and Dunnett's multiple‐comparison post tests or Student's *t*‐test, as appropriate, with GraphPad Prism v5.04. * = *p* <0.05, *** = *p* < 0.001.

## Conflict of interest

The authors declare no financial or commercial conflict of interest.

**Abbreviations**
BCG: Bacillus Calmette–Guérin
BMM: bone marrow‐derived macrophage
CTLR: C‐type lectin receptor
CTLD: C‐type lectin‐like domain
KLH: keyhole limpet hemocyanin
TDM: trehalose dimycolate

## Supporting information

As a service to our authors and readers, this journal provides supporting information supplied by the authors. Such materials are peer reviewed and may be re‐organized for online delivery, but are not copy‐edited or typeset. Technical support issues arising from supporting information (other than missing files) should be addressed to the authors.

Supporting Figures

Peer Review Correspondence
