# Supplementary material for: Mycobacterial receptor, Clec4d (CLECSF8, MCL), is coregulated with Mincle and upregulated on mouse myeloid cells following microbial challenge
Source: Eur J Immunol. 2015 Dec 8;46(2):381–9. doi: 10.1002/eji.201545858 (PMC4833188; doi:10.1002/eji.201545858)
Supplement: Supplementary file 1 — Supporting Figures [file EJI-46-381-s001.pdf]

European Journal of Immunology

Supporting Information
for
DOI 10.1002/eji.201545858

Bernhard Kerscher, Gillian J. Wilson, Delyth M. Reid, Daiki Mori, Julie A. Taylor, Gurdyal S. Besra, Sho Yamasaki, Janet A. Willment and Gordon D. Brown

Mycobacterial receptor, Clec4d (CLECSF8, MCL), is coregulated with Mincle and upregulated on mouse myeloid cells following microbial challenge

© 2015 The Authors. *European Journal of Immunology* published by WILEY-VCH Verlag GmbH & Co. KGaA, Weinheim.

Supplemental Data

Figure S1

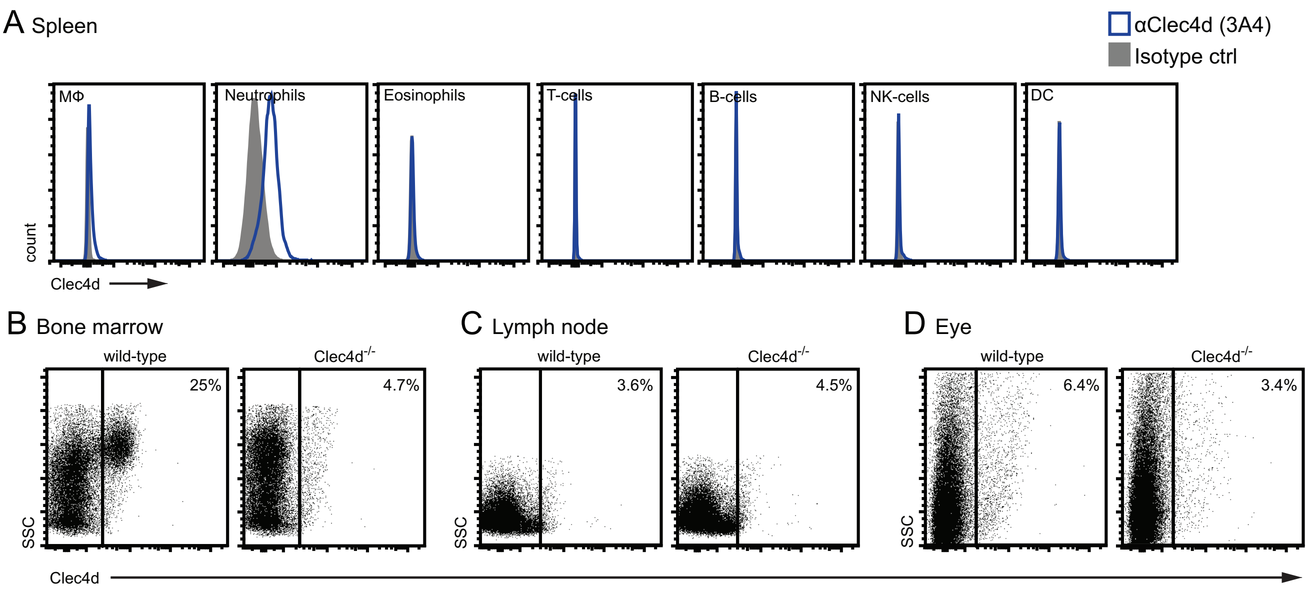

Supplementary Figure 1. Clec4d expression on (A) spleen, (B) bone marrow, (C) lymph node and (D) eye cells assessed by flow cytometry. Grey, isotype; blue, anti-mClec4d (3A4). Data representative of at least 2 mice. See also Fig. 2.

Figure S2

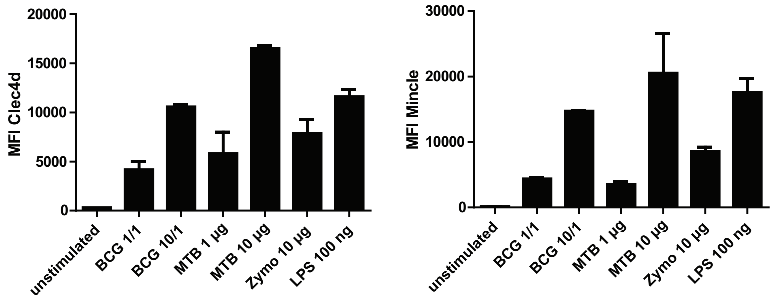

Supplementary Figure 2. Effect of microbial stimulation on bone-marrow macrophages. Bone marrow-derived macrophages were treated with microbial stimuli (BCG, *M. bovis* BCG; MTB, *M. tuberculosis*; Zymo, Zymosan; LPS, lipopolysaccharide) as indicated for 16 h followed by flow cytometry analysis for expression of Clec4d and Mincle. Data shows a single experiment.

Figure S3

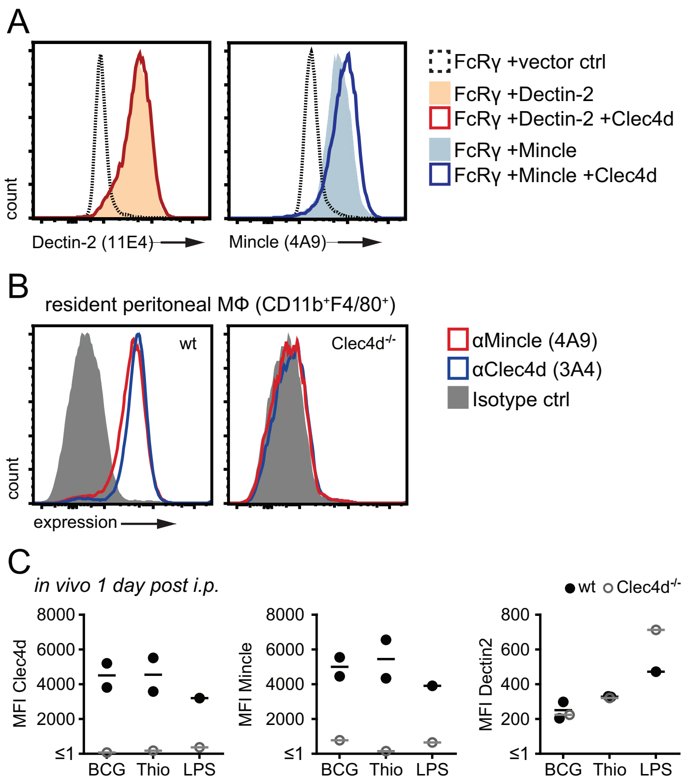

Supplementary Figure 3. Surface expression of Mincle and Clec4d are interdependent. (A) Total protein expression related to Figure 4A. NIH-3T3 retrovirally transduced with FcRγ, Clec4d and Dectin-2 (11E4) or Mincle (4A9) were fixed (1% formaldehyde) and permeabilised (0.5% saponin) before staining for Dectin-2 and Mincle and analysis by flow cytometry. Representative of two experiments. (B) Freshly collected peritoneal cells from wild-type or Clec4d$^{-/-}$ mice were stained for Clec4d (3A4) or Mincle (4A9) and analysed by flow cytometry. (C) Wild-type or Clec4d$^{-/-}$ mice were injected i.p. with the indicated immunomodulators and expression of Clec4d (3A4), Mincle (4A9) and Dectin-2 (11E4) analysed one day post injection by flow cytometry. Data from a single experiment.

Figure S4

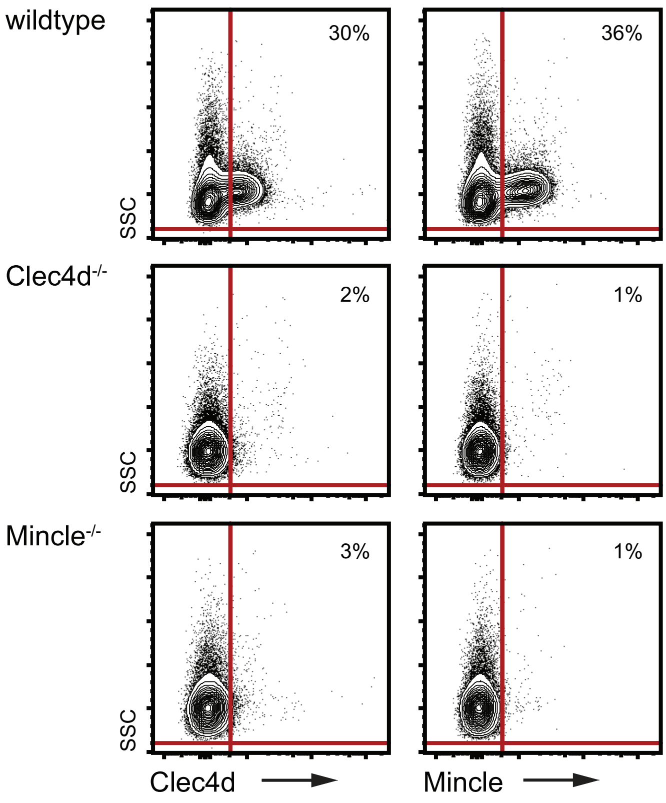

Supplementary Figure 4. Surface expression of Clec4d and Mincle on naïve cells are interdependent. Freshly collected bone marrow from wild-type, Clec4d$^{-/-}$ and Mincle$^{-/-}$ mice was stained for Clec4d (3A4) or Mincle (4A9) and analysed by flow cytometry. Plots show receptor expression on CD45^{+} CD11b^{+} cells. Data from a single experiment.

Figure S5

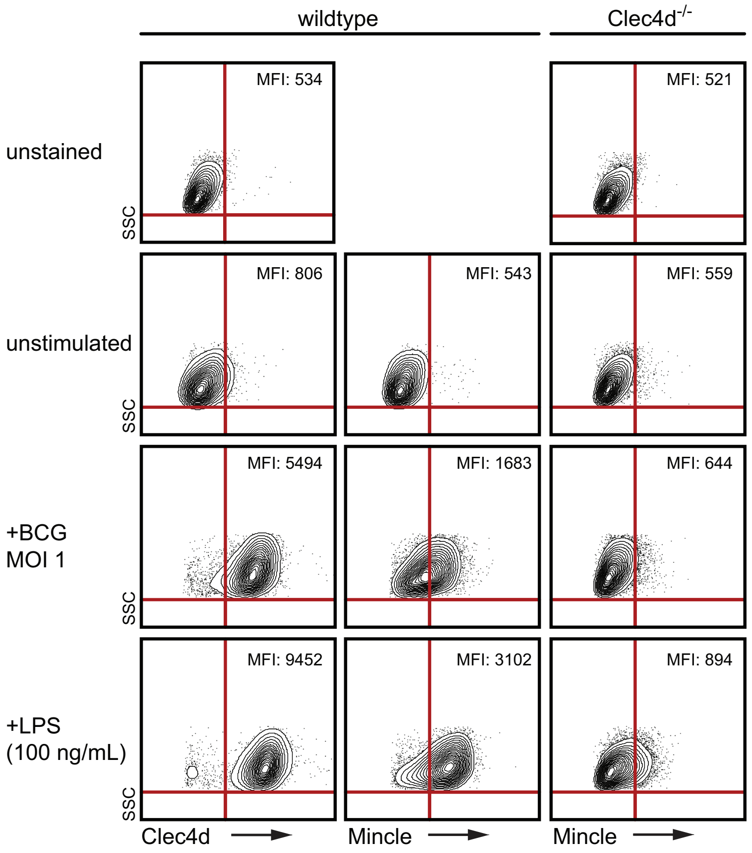

Supplementary Figure 5. Surface expression of Mincle is dependent on the presence of Clec4d. Bronchoalveolar lavage cells from naïve wild-type or Clec4d$^{-/-}$ mice (pool of 10 mice each) were plated and stimulated as indicated for 16 h. Expression of Clec4d (3A4) and Mincle (4A9) was then analysed by flow cytometry. Data from a single experiment.
