# Supplementary material for: Mycobacterial receptor, Clec4d (CLECSF8, MCL), is coregulated with Mincle and upregulated on mouse myeloid cells following microbial challenge
Source: Eur J Immunol. 2015 Dec 8;46(2):381–9. doi: 10.1002/eji.201545858 (PMC4833188; doi:10.1002/eji.201545858)
Supplement: Supplementary file 2 — Peer Review Correspondence [file EJI-46-381-s002.pdf]

Peer review correspondence

The mycobacterial receptor, Clec4d (CLECSF8, MCL) is co-regulated with Mincle and upregulated on mouse myeloid cells following microbial challenge

Bernhard Kerscher, Gillian J. Wilson, Delyth M. Reid, Daiki Mori, Julie A. Taylor, Gurdyal S. Besra, Sho Yamasaki, Janet A. Willment and Gordon D. Brown

Corresponding author: Janet A. Willment, Institute of Medical Sciences, University of Aberdeen, Foresterhill, Aberdeen, United Kingdom

---

Review Timeline:	Submission date:	11 June 2015
	First editorial decision:	3 July 2015
	Revision received:	15 September 2015
	Accepted:	6 November 2015

Handling Executive Committee member: Dr. Steffen Jung

Please note that the correspondence below does not include the standard editorial instructions regarding preparation and submission of revised manuscripts, only the scientific revisions requested and addressed.

First Editorial Decision – 3 July 2015

Dear Dr. Willment,

Manuscript ID eji.201545858 entitled "The mycobacterial receptor, Clecsf8 (CLEC4D) requires Mincle for surface expression and is upregulated on myeloid cells following microbial challenge." which you submitted to the European Journal of Immunology has been reviewed. The comments of the referees are included at the bottom of this letter.

You will see that the reviewers missed the novelty of your study. Therefore please sharpen the Statement of this study, be more precise concerning the nomenclature,clarify the Mincl dependence and better highlight the importance of this study.

A revised version of your manuscript that takes into account the comments of the referees will be reconsidered for publication. Should you disagree with any of the referees' concerns, you should address

European Journal of Immunology

this in your point-by-point response and provide solid scientific reasons for why you will not make the requested changes.

You should also pay close attention to the editorial comments included below. **In particular, please edit your figure legends to follow Journal standards as outlined in the editorial comments. Failure to do this will result in delays in the re-review process.**

Please note that submitting a revision of your manuscript does not guarantee eventual acceptance, and that your revision will be re-reviewed by the referees before a decision is rendered.

If the revision of the paper is expected to take more than three months, please inform the editorial office. Revisions taking longer than six months may be assessed by new referees to ensure the relevance and timeliness of the data.

Once again, thank you for submitting your manuscript to European Journal of Immunology and we look forward to receiving your revision.

Yours sincerely,
Katharina Schmidt

On behalf of Dr. Steffen Jung

Dr. Katharina Schmidt
Editorial Office
European Journal of Immunology
e-mail: ejied@wiley.com
www.eji-journal.eu

****

Reviewer: 1

Comments to the Author
The present study provides some additional information relevant to ongoing confusion / controversy regarding the role of the macrophage receptor Clecsf8, confirming studies showing that in the presence of mincle, there is increased cell surface expression of Clecsf8 and vice versa. However, there are some fundamental issues:

European Journal of Immunology

[1] The data do not support the first statement in the title: "The mycobacterial receptor, Clecsf8 ... requires mincle for surface expression". The issue in interpretation is highlighted on page 8, where it is stated that "FcRgamma alone was able to promote moderate levels of Clecsf8 surface expression" and then, following evidence for increased expression in the presence of mincle, the conclusion is made that "This shows that Clecsf8 requires mincle ... for expression at the cell surface". The latter statement directly contradicts what was just stated and ends up in the potentially misleading title.

[2] Results from another group, reported in reference 6, are interpreted as demonstrating that Clecsf8 [Dectin-3] forms a complex with Dectin-2. The results reported here and in previous work from the authors of this manuscript are interpreted as evidence against formation of such a complex, but there is no effort made to explain why different results are obtained.

[3] In previous work from some of the authors of this manuscript, reported in reference 17, the distinction between expression of Clecsf8 and mincle at the mRNA and protein levels and appearance of detectable protein at the cell surface was investigated. The present study refers to movement to the cell surface as a mechanism for controlling the surface expression, but the underlying mechanism is not directly investigated as in the previous more detailed study, since only surface expression on the isolated cell types is measured here. Mechanistic investigated is left to "future work" in the final sentence of the Discussion.

In addition to these major substantive issues, the manuscript is not in good condition. For example, there is little consistency in defining or use of abbreviations. Those used without explanation include CTLD, KLH, BCG, BSA, PBL, LPS, GFP, ADC, bio, and TB [usually used as an abbreviation for the disease tuberculosis, where in fact Mycobacterium tuberculosis seems to be meant here]. In addition, C-type lectin receptors are referred to variously as CTLs and CTLRs and bone marrow-derived macrophages are both BMM and BMDM. The introduction suggests that all of the Dectin-2-like receptors associate with FcRgamma, in the statement that "Like the other Dectin-2 family members, surface expression of Clecsf8 requires association with the signaling adapter FcRgamma", when no such association of DCIR has been demonstrated. Figure 1C is unconvincing as a demonstration of specificity of the monoclonal antibodies: there are no molecular weight markers and small portions of the gels/blots are shown.

Regarding nomenclature, the protein referred to in this manuscript as Clecsf8 is commonly referred to as macrophage C-type lectin or MCL and this was the name originally applied to it. This is the name used in some papers by these authors as well, but it appears only once in this manuscript and not at all in the abstract, which would make the work very hard for others to relate to previously published work. The Clecsf8 name used here does not appear in the title of manuscripts from any other group in the past 10 years.

European Journal of Immunology

Reviewer: 2

Comments to the Author

The research groups of Gordon Brown and Sho Yamasaki have been responsible for a large proportion of the research over the last few years in this burgeoning field. Here they have developed new antibodies to mouse clecsf8 and use these to analyze receptor expression. They confirm previous studies in the rat showing that Mincle and clecsf8 associate. At the same time they are unable to confirm the previously published interaction between Clecsf8 and Dectin-2. This is an important finding. There are some minor problems that should be addressed before the article can be considered for publication:

1. Abstract, line 30: The data do not support the claim that Clecsf8 is strongly upregulated on pulmonary myeloid cells following BCG infection (see also point 9).
2. Introduction, line 40: The Dectin-2 gene cluster lies upstream of the NK complex, and not within it. For an overview of the NK complex see Kelley, J., Walter, L., & Trowsdale, J. (2005). Comparative Genomics of Natural Killer Cell Receptor Gene Clusters. PLoS Genetics, 1(2), e27.
3. Introduction line 58, reference 4 cited here should probably be reference 9. There are at least 2 other instances where these two citations have been confused.
4. Results, figure 1C. This figure should include MW markers.
5. Results, line 118, figure 2D. Clecsf8 is clearly expressed on alveolar neutrophils. The claim that clecsf8 is expressed at low levels on alveolar macrophages is debatable. At best it could be said that there is marginal expression by a small subset of these macrophages. The authors might equally claim that clecsf8 is expressed on eosinophils. The text should be updated.
6. Results figure S1D, to me the wild-type seems to have a higher number of positive cells than the knockout. This figure should include numbers to indicate the percent positive cells in each FACS plot, because it’s difficult for me to count all the dots. It’s also unclear to me what eye cells are. The information on cell isolation should be included in the methods.
7. Results, figure 3C – E. It is unclear what the fold-change represents. Is this fold-change from the cells in figure 3B? Hopefully not because the expression there is essentially zero, making fold-change meaningless. It should be expressed as fold-change from the expression in figure 3A, since this is the starting point, and this information should be included somewhere.
8. Results, Figure 3C. The letters and numbers used to label the bars need to be described in the figure legend. I googled “Cay CaH” and got pictures of some fabulous-looking Indonesian food. I suspect that this wasn’t what the authors used to stimulate their cells.
9. Results, figure 5. This figure lacks statistical analysis, and needs it since the differences are relatively small. Figure 5B is misleading. The white bars have an MFI of 500, but these correspond to an expression of zero since they are knockout for clecsf8. In this context, the increase in expression seen after BCG treatment of alveolar macrophages may be statistically significant, but it is a question of whether or not it is biologically significant since it is very low expression. This data should be shown as FACS plots so that

European Journal of Immunology

the real expression difference is more apparent. This upregulation is surprisingly low, given the proposed role of clecsf8 in defense against mycobacteria. This point should be discussed more in the text.

Reviewer: 3

Comments to the Author
In their study, Kerscher and colleagues analyzed the expression of the C-type lectin receptor Clecsf8 (MCL) under different conditions in vitro and in vivo. MCL was predominantly expressed on myeloid cells. Basal expression on alveolar cells was rather low but was upregulated after infection with Mycobacterium bovis BCG. In vitro MCL expression on macrophages was induced by various microbial stimuli but not by cytokines. MCL expression required the presence of FcRgamma and Mincle and both, the expression of MCL and Mincle, are mutually dependent. From these data the authors conclude, that MCL is an inducible C-type lectin receptor on myeloid cells linked to the expression of Mincle.

General comments
This is an interesting study that analyses the regulation of MCL expression in the context of myeloid cells. However, to me it is not surprising that MCL is induced on myeloid cells by various TLR ligands. Moreover, the manuscripts lacks to some extend accuracy on the conclusion that MCL and Mincle expression are mutually dependent and on M. bovis BCG as a surrogate for mycobacterial infections.

1. Based on results presented in Figure 5, the authors state that in contrast to wildtype mice “infection of (MCL) knockout animals with BCG did not lead to an increase expression of Mincle” and that ”expression of Dectin-2 was unaltered following BCG infection in wildtype and MCL-deficient mice” and conclude that the expression of MCL is linked to that of Mincle but not to that of Dectin-2. Although the in vitro data presented in this manuscript fully support this, the in vivo data are not that strong at all. This is highlighted by the fact, that statistical analysis was performed in Figure 4 whereas no significant differences are presented in Figure 5. Only if (a) there is a significant induction of either receptor after infection and if (b) the expression after infection is significantly different the conclusion made by the authors is justified. Otherwise, an apparent discrepancy between in vitro and in vivo findings has to be discussed.
2. It is important to differentiate between M. bovis BCG and Mycobacterium tuberculosis. Either pathogen could not serve as a surrogate for “mycobacterial infection”. This is especially highlighted by the different outcome of M. bovis BCG (Behler et al. 2012) and M. tuberculosis (Heitmann et al. 2012; not cited in the present manuscript) infection in Mincle-deficient mice. Hence, the statement in line 199/200 is wrong that “Mincle-deficiency led to increased mycobacterial lung burdens”. This holds only true for M. bovis BCG infection but not for aerosol infection with M. tuberculosis. The authors should strictly differentiate between these two pathogens and no generalized conclusion should be drawn.

Specific comments

3. Figure 3A and B; are these histograms from one experiment? Figure 3B lacks a positive control.

4. Figure 4A; what is the actual expression of FcRgamma, Mincle and Dectin-2 in the transduced cells?

First revision – authors' response – 15 September 2015

Reviewer: 1

[1] The data do not support the first statement in the title: "The mycobacterial receptor, Clecsf8 ... requires mincle for surface expression". The issue in interpretation is highlighted on page 8, where it is stated that "FcRgamma alone was able to promote moderate levels of Clecsf8 surface expression" and then, following evidence for increased expression in the presence of mincle, the conclusion is made that "This shows that Clecsf8 requires mincle ... for expression at the cell surface". The latter statement directly contradicts what was just stated and ends up in the potentially misleading title.

Our Response: We have changed the title as requested, although we now provide additional data (Figure 4D&E and S4) demonstrating that Clecsf8 expression is absent on Mincle-deficient primary cells from naïve mice and that Mincle is required for upregulation of Clecsf8 surface expression upon microbial stimuli on BMM. The rather low basal Clecsf8-expression in the presence of FcRg alone might be a result of protein overexpression in our transduced cell line.

[2] Results from another group, reported in reference 6, are interpreted as demonstrating that Clecsf8 [Dectin-3] forms a complex with Dectin-2. The results reported here and in previous work from the authors of this manuscript are interpreted as evidence against formation of such a complex, but there is no effort made to explain why different results are obtained.

Our Response: We are currently collaborating with the authors of that study to determine the cause of these differences between the labs. While our results strongly support the idea of a Clecsf8-Mincle interaction, we find no evidence of a similar interdependence of Dectin-2 and Clecsf8.

[3] In previous work from some of the authors of this manuscript, reported in reference 17, the distinction between expression of Clecsf8 and mincle at the mRNA and protein levels and appearance of detectable protein at the cell surface was investigated. The present study refers to movement to the cell surface as a mechanism for controlling the surface expression, but the underlying mechanism is not directly investigated as in the previous more detailed study, since only surface expression on the isolated cell types is measured here. Mechanistic investigated is left to "future work" in the final sentence of the Discussion.

Our Response: We are currently investigating the mechanism behind the induction of Clecsf8 surface expression. However, we do not feel that such studies are within the scope of the current submission. Our

European Journal of Immunology

objective here is to characterise expression on primary cells and ex vivo, the changes that occur during infection and to show that expression is linked to that of Mincle.

In addition to these major substantive issues, the manuscript is not in good condition. For example, there is little consistency in defining or use of abbreviations. Those used without explanation include CTLD, KLH, BCG, BSA, PBL, LPS, GFP, ADC, bio, and TB [usually used as an abbreviation for the disease tuberculosis, where in fact Mycobacterium tuberculosis seems to be meant here]. In addition, C-type lectin receptors are referred to variously as CTLs and CTLRs and bone marrow-derived macrophages are both BMM and BMDM. The introduction suggests that all of the Dectin-2-like receptors associate with FcRgamma, in the statement that "Like the other Dectin-2 family members, surface expression of Clecsf8 requires association with the signaling adapter FcRgamma", when no such association of DCIR has been demonstrated. Figure 1C is unconvincing as a demonstration of specificity of the monoclonal antibodies: there are no molecular weight markers and small portions of the gels/blots are shown.
Our Response: We have clarified abbreviations and also included MW markers. We would like to highlight that we are probing purified recombinant Fc-tagged proteins which are larger than the native receptor proteins due to the Fc-tag. The sole purpose of Figure 1C was to demonstrate the functionality of our new Clecsf8 mABs in western blot under reducing and non-reducing conditions. FACS staining of wild-type and Clecsf8-deficient PBL (now figure 1 G) were used to assess specificity. However, we have now also included a new Figure (1F) probing wild-type and Clecsf8-/- BMM cell lysates to demonstrate specificity of mAB 3A4 in western blot.

Regarding nomenclature, the protein referred to in this manuscript as Clecsf8 is commonly referred to as macrophage C-type lectin or MCL and this was the name originally applied to it. This is the name used in some papers by these authors as well, but it appears only once in this manuscript and not at all in the abstract, which would make the work very hard for others to relate to previously published work. The Clecsf8 name used here does not appear in the title of manuscripts from any other group in the past 10 years.
Our Response: We have added MCL to the abstract as suggested. We also changed the name to its more commonly used systematic name Clec4d throughout the manuscript, as requested.

Reviewer: 2

Comments to the Author
The research groups of Gordon Brown and Sho Yamasaki have been responsible for a large proportion of the research over the last few years in this burgeoning field. Here they have developed new antibodies to mouse clecsf8 and use these to analyze receptor expression. They confirm previous studies in the rat

European Journal of Immunology

showing that Mincle and clecsf8 associate. At the same time they are unable to confirm the previously published interaction between Clecsf8 and Dectin-2. This is an important finding. There are some minor problems that should be addressed before the article can be considered for publication:

1. Abstract, line 30: The data do not support the claim that Clecsf8 is strongly upregulated on pulmonary myeloid cells following BCG infection (see also point 9).

Our Response: We made the appropriate change in the text.

2. Introduction, line 40: The Dectin-2 gene cluster lies upstream of the NK complex, and not within it. For an overview of the NK complex see Kelley, J., Walter, L., & Trowsdale, J. (2005). Comparative Genomics of Natural Killer Cell Receptor Gene Clusters. PLoS Genetics, 1(2), e27.

Our Response: We made the appropriate change in the text.

3. Introduction line 58, reference 4 cited here should probably be reference 9. There are at least 2 other instances where these two citations have been confused.

Our Response: We made the appropriate change in the text.

4. Results, figure 1C. This figure should include MW markers.

Our Response: We now have included MW markers, as requested.

5. Results, line 118, figure 2D. Clecsf8 is clearly expressed on alveolar neutrophils. The claim that clecsf8 is expressed at low levels on alveolar macrophages is debatable. At best it could be said that there is marginal expression by a small subset of these macrophages. The authors might equally claim that clecsf8 is expressed on eosinophils. The text should be updated.

Our Response: We made the appropriate change in the text.

6. Results figure S1D, to me the wild-type seems to have a higher number of positive cells than the knockout. This figure should include numbers to indicate the percent positive cells in each FACS plot, because it's difficult for me to count all the dots. It's also unclear to me what eye cells are. The information on cell isolation should be included in the methods.

Our Response: We included quantitative measures in the figures and made the appropriate changes in the text

7. Results, figure 3C – E. It is unclear what the fold-change represents. Is this fold-change from the cells in figure 3B? Hopefully not because the expression there is essentially zero, making fold-change meaningless. It should be expressed as fold-change from the expression in figure 3A, since this is the starting point, and this information should be included somewhere.

Our Response: Our methods section includes a description of the "fold-change" calculation, and we have now clarified this further in the text and figure legends.

European Journal of Immunology

8. Results, Figure 3C. The letters and numbers used to label the bars need to be described in the figure legend. I googled “Cay CaH” and got pictures of some fabulous-looking Indonesian food. I suspect that this wasn’t what the authors used to stimulate their cells.
Our Response: We have clarified abbreviations used within the manuscript, figures and figure legends.

9. Results, figure 5. This figure lacks statistical analysis, and needs it since the differences are relatively small. Figure 5B is misleading. The white bars have an MFI of 500, but these correspond to an expression of zero since they are knockout for clecsf8. In this context, the increase in expression seen after BCG treatment of alveolar macrophages may be statistically significant, but it is a question of whether or not it is biologically significant since it is very low expression. This data should be shown as FACS plots so that the real expression difference is more apparent. This upregulation is surprisingly low, given the proposed role of clecsf8 in defense against mycobacteria. This point should be discussed more in the text.
Our Response: We have now included statistical analysis and a FACS plot to visualise the significant expression on alveolar macrophages. We have added more discussion on this point in the text.

Reviewer: 3

Comments to the Author
In their study, Kerscher and colleagues analyzed the expression of the C-type lectin receptor Clecsf8 (MCL) under different conditions in vitro and in vivo. MCL was predominantly expressed on myeloid cells. Basal expression on alveolar cells was rather low but was upregulated after infection with Mycobacterium bovis BCG. In vitro MCL expression on macrophages was induced by various microbial stimuli but not by cytokines. MCL expression required the presence of FcRgamma and Mincle and both, the expression of MCL and Mincle, are mutually dependent. From these data the authors conclude, that MCL is an inducible C-type lectin receptor on myeloid cells linked to the expression of Mincle.

General comments
This is an interesting study that analyses the regulation of MCL expression in the context of myeloid cells. However, to me it is not surprising that MCL is induced on myeloid cells by various TLR ligands. Moreover, the manuscripts lacks to some extend accuracy on the conclusion that MCL and Mincle expression are mutually dependent and on M. bovis BCG as a surrogate for mycobacterial infections.

1. Based on results presented in Figure 5, the authors state that in contrast to wildtype mice “infection of (MCL) knockout animals with BCG did not lead to an increase expression of Mincle” and that ”expression of Dectin-2 was unaltered following BCG infection in wildtype and MCL-deficient mice” and conclude that the expression of MCL is linked to that of Mincle but not to that of Dectin-2. Although the in vitro data

European Journal of Immunology

presented in this manuscript fully support this, the in vivo data are not that strong at all. This is highlighted by the fact, that statistical analysis was performed in Figure 4 whereas no significant differences are presented in Figure 5. Only if (a) there is a significant induction of either receptor after infection and if (b) the expression after infection is significantly different the conclusion made by the authors is justified. Otherwise, an apparent discrepancy between in vitro and in vivo findings has to be discussed.
Our Response: We now include statistical analysis for this experiment, as requested.

2. It is important to differentiate between M. bovis BCG and Mycobacterium tuberculosis. Either pathogen could not serve as a surrogate for “mycobacterial infection”. This is especially highlighted by the different outcome of M. bovis BCG (Behler et al. 2012) and M. tuberculosis (Heitmann et al. 2012; not cited in the present manuscript) infection in Mincle-deficient mice. Hence, the statement in line 199/200 is wrong that “Mincle-deficiency led to increased mycobacterial lung burdens”. This holds only true for M. bovis BCG infection but not for aerosol infection with M. tuberculosis. The authors should strictly differentiate between these two pathogens and no generalized conclusion should be drawn.
Our Response: We made the appropriate changes in the text.
Specific comments

3. Figure 3A and B; are these histograms from one experiment? Figure 3B lacks a positive control.
Our Response: These histograms are representative of >3 experiments. Figure 3B visualises receptor expression of the unstimulated control within the following panels. We have clarified this in the text.

4. Figure 4A; what is the actual expression of FcRgamma, Mincle and Dectin-2 in the transduced cells?
Our Response: We now show total protein expression levels of Mincle and Dectin-2 by western blot (Fig. 4A) and FACS staining of fixed-permeabilised cells (Fig. S3A).

Second Editorial Decision – 14 October 2015

Dear Dr. Willment,

It is a pleasure to provisionally accept your manuscript entitled "The mycobacterial receptor, Clec4d (CLECSF8, MCL) is co-regulated with Mincle and upregulated on myeloid cells following microbial challenge." for publication in the European Journal of Immunology. For final acceptance, please follow the instructions below and return the requested items as soon as possible as we cannot process your manuscript further until all items listed below are dealt with.

Please note that EJI articles are now published online a few days after final acceptance (see Accepted Articles: http://onlinelibrary.wiley.com/journal/10.1002/(ISSN)1521-4141/accepted). The files used for the

European Journal of Immunology

Peer review correspondence

Accepted Articles are the final files and information supplied by you in Manuscript Central. You should therefore check that all the information (including author names) is correct as changes will NOT be permitted until the proofs stage.

We look forward to hearing from you and thank you for submitting your manuscript to the European Journal of Immunology.

Yours sincerely,
Karen Chu

on behalf of Dr. Steffen Jung

Dr. Karen Chu
Editorial Office
European Journal of Immunology
e-mail: ejied@wiley.com
www.eji-journal.eu
